# Accuracy of currently available neonatal respiratory function monitors for neonatal resuscitation

**DOI:** 10.1007/s00431-016-2739-1

**Published:** 2016-06-08

**Authors:** Charlotte Verbeek, Henriëtte A. van Zanten, Jeroen J. van Vonderen, Marcus J Kitchen, Stuart B. Hooper, Arjan B. te Pas

**Affiliations:** Division of Neonatology, postzone J6-S, Leiden University Medical Centre, Albinusdreef 2, 2333ZA Leiden, The Netherlands; School of Physics and Astronomy, Monash University, Melbourne, Victoria Australia; MIMR-PHI Institute for Medical Research, Monash University, Clayton, Victoria Australia

**Keywords:** Neonatology, Respiratory function monitor, Resuscitation, Tidal volume

## Abstract

This study aimed to test the accuracy in volume measurements of three available respiratory function monitors (RFMs) for neonatal resuscitation and the effect of changing gas conditions. The Florian, New Life Box Neo-RSD (NLB Neo-RSD) and NICO RFM were tested on accuracy with volumes of 10 and 20 mL and on changes in volume measurements under changing gas conditions (oxygen level 21–100 % and from cold dry air (24 ± 2 °C) to heated humidified air (37 °C). Volume differences >10 % were considered clinically relevant. We found that the mean (SD) volume difference was clinically acceptable for all devices (10, 20 mL): Florian (+8.4 (1.2)%, +8.4 (0.5)%); NLB Neo-RSD (+5.8 (1.1)%, +4.3 (1.4)%); and NICO (−8.2 (0.9)%, −8.7 (0.8)%). Changing from cold dry to heated humidified air increased the volume difference using the Florian (cold dry air, heated humidified air (+5.2 (1.2)%, +12.2 (0.9)%) but not NLB Neo-RSD (+2.0(1.6)%, +3.4(2.8)%) and NICO (−2.3 % (0.8), +0.1 (0.6)%). Similarly, when using heated humidified air, increasing oxygen enlarged increased the volume difference using the Florian (oxygen 21 %, 100 %: +12.2(1.0)%, +19.8(1.1)%), but not NLB Neo-RSD (+0.2(1.9)%, +1.1(2.8)%) and NICO (−5.6(0.9)%, −3.7(0.9)%). Clinically relevant changes occurred when changing both gas conditions (Florian +25.7(1.7)%; NLB Neo-RSD +3.8(2.4)%; NICO −5.7(1.4)%).

*Conclusion*: The available RFMs demonstrated clinically acceptable deviations in volume measurements, except for the Florian when changing gas conditions.

**Table:** 

**What is known:**
•*Respiratory function monitors (RFMs) are increasingly used for volume measurements during respiratory support of infants at birth.* •*During respiratory support at birth, gas conditions can change quickly, which can influence the volume measurements.*
**What is new:**
•*The available RFMs have clinically acceptable deviations when measuring the accuracy of volume measurements*. •*The RFM using a hot wire anemometer demonstrated clinically relevant deviations in volume measurements when changing the gas conditions. These deviations have to be taken into account when interpreting the volumes directly at birth.*

## Introduction

Adequate mask ventilation is the cornerstone of neonatal resuscitation at birth [1, 2]. However, mask ventilation is very difficult, requiring refined skills and experienced staff, and is often hampered by large mask leak and airway obstruction [3]. In addition, the lungs of preterm infants are prone to injury and volume trauma can easily occur when the caregiver is unaware of the volumes being given during pressure controlled ventilation [1, 4–6]. While current guidelines do not recommend the use of a respiratory function monitor (RFM) during neonatal resuscitation at birth [6], it has been suggested that RFMs may improve mask ventilation, leak, and technique, and measuring tidal volumes has the potential to decrease lung injury [2, 7–12]. As a result, a large multicentre randomized trial is currently being undertaken (monitor trial, trial number NTR4104) to investigate whether a RFM will improve the tidal ventilation given to preterm infants at birth.

A RFM measures the pressures given and gas flow into and out of the lungs from which inflation and expired volumes are calculated. Currently, three types of RFMs are available for measuring lung function at birth using different techniques to measure gas flow and software for calculations of the tidal volume. The most commonly used device in the delivery room is the Florian Neonatal RFM (Acutronic Medical Systems AG, Hirzel, Switzerland) using a hotwire anemometer [10, 13–15]. Other studies have used a Respironics Novametrix non-invasive cardiac output (NICO) monitor (Novametrix Medical Systems Inc., Wallingford, Connecticut), which uses a differential pressure pneumotachometer with a fixed orifice [8]. In the monitor trial, a newly developed resuscitation monitor is used with a built-in New Life Box Neo-RSD (NLB Neo-RSD, Advanced Life Diagnostics UG, Weener, Germany) for lung function measurements, using a differential pressure pneumotachometer with a variable orifice.

Studies comparing types of RFM have been performed [5, 16–20], but so far there is no study available comparing the three available RFMs for monitoring neonatal resuscitation in the delivery room. During neonatal resuscitation, the oxygen content of the inspired gas can quickly change from 21 to 100 % O_2_ depending upon the infant’s need. In addition, heated humidified air is increasingly used for ventilation in preterm infants at birth, which will influence density gas [6, 21, 22]. However, depending on how much preparation time was available, the temperature and humidity may still be increasing while the infant is being ventilated. Modern ventilators using distal flow probes for measurements correct for these differences in gas condition, but currently RFMs used in the delivery room do not correct for this. It is unclear how much this will influence the gas flow measurements of the different RFMs. The manufacturers’ data on accuracy are obtained under the most ideal gas conditions and are not comparable to clinical practice in the delivery room.

The objective of this study was to compare the accuracy of current available RFMs and to test the effect of changing gas conditions on the volume measurements.

## Materials and methods

This bench top study was performed at the Neonatal Intensive Care Unit of the Leiden University Medical Centre, the Netherlands. No patients or participants were involved, approval of the Institutional Review Board of our hospital was not necessary.

We compared three different types of RFMs: (1) The Florian Neonatal RFM (Acutronic Medical Systems AG, Hirzel, Switzerland), using a hotwire anemometer with a dead space of approximately 1 mL and according to the manufacturer an accuracy of ±8 %. The anemometer measures the gas flow rate by measuring resistance changes within the hotwire caused by gas flow; (2) a New Life Box Neo-RSD (NLB Neo-RSD, Advanced Life Diagnostics UG, Weener, Germany). This device uses a variable orifice pneumotachometer (Avea Varflex Flow Transducer, CareFusion, Yorba Linda, USA) connected to the NLB Neo-RSD and has an accuracy of ±5 % (manufacturer’s data) and a dead space of 0.7 mL; and (3) Respironics Novametrix Non-Invasive Cardiac Output (NICO) monitor (Novametrix Medical Systems Inc., Wallingford, Connecticut). This device uses a fixed orifice pneumotachometer which determines the gas flow rate by measuring the pressure difference across a resistance, has a dead space of 1 mL and a flow accuracy of ±3 % (manufacturer’s data).

Each RFM was calibrated according to manufacturer’s instructions immediately prior to every performed measurement.

### Accuracy

**Fig. 1:**
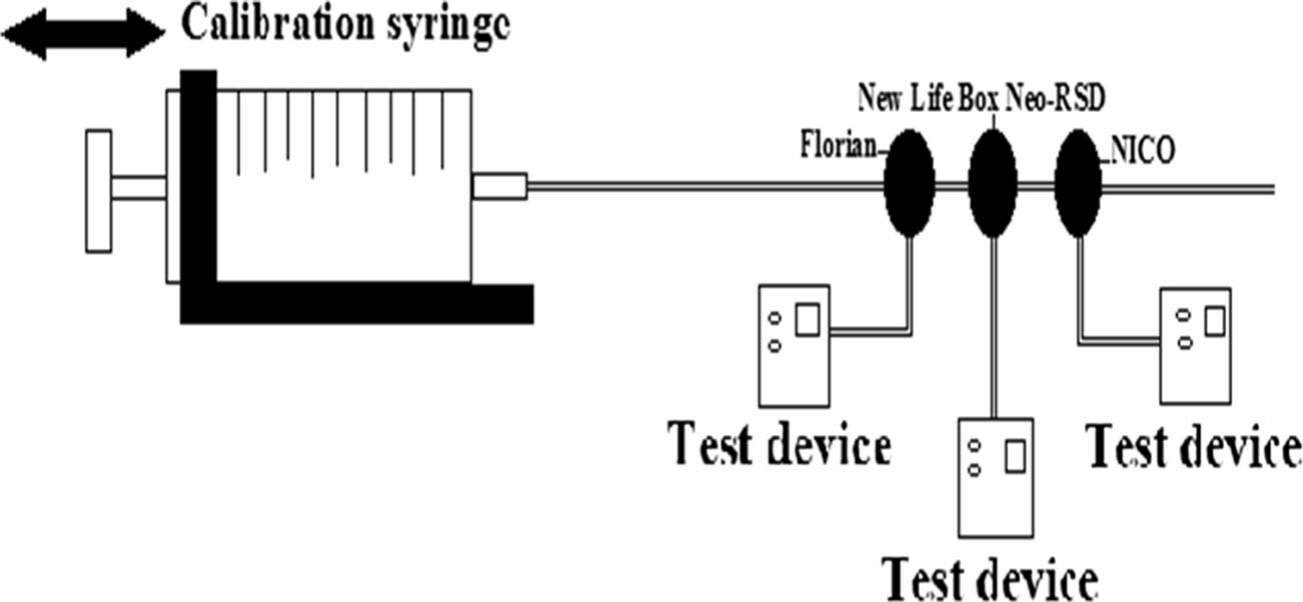
The experimental set-up to determine the accuracy of the three RFMs

To compare the accuracy of the three types of RFM, we tested the devices by using a known volume and compared this with the volume measured by the RFM. To ventilate with a known volume, we used a glass syringe (Fortuna Optima Ganzglasspritze, Luer Glas Tip 7.102–41, Poulten & Graf GmbH., Wertheim, Germany) with adjustable stops. The glass syringe was placed in a purpose built framework and driven manually between the stop positions, which made it possible to accurately ventilate with the known volumes of 10.0 and 20.0 mL (Fig. 1). The accuracy of the volume of the glass syringe has been tested using an industry standard BioTek VT plus gas flow analyzer (BioTek Instruments, Winooski, VT, USA). For the experiment two different flow probes were used for each device, and ten consecutive measurements were carried out for both volumes. The glass syringe was connected to the sensors of the three RFMs in a serial order, which formed our basic set up (Fig. 1). Data collection for both the Florian and the NICO RFM was performed using the Spectra physiological recording program (Grove Medical Limited, Hampton/London, UK); data was recorded at 200 Hz. Data collection for the NLB Neo-RSD was performed using the Polybench physiological recording program (Applied Biosignals GmbH edition 1.30.0.3505, Weener, Germany).

### Differences in volume measurements during changing gas conditions

**Fig. 2:**
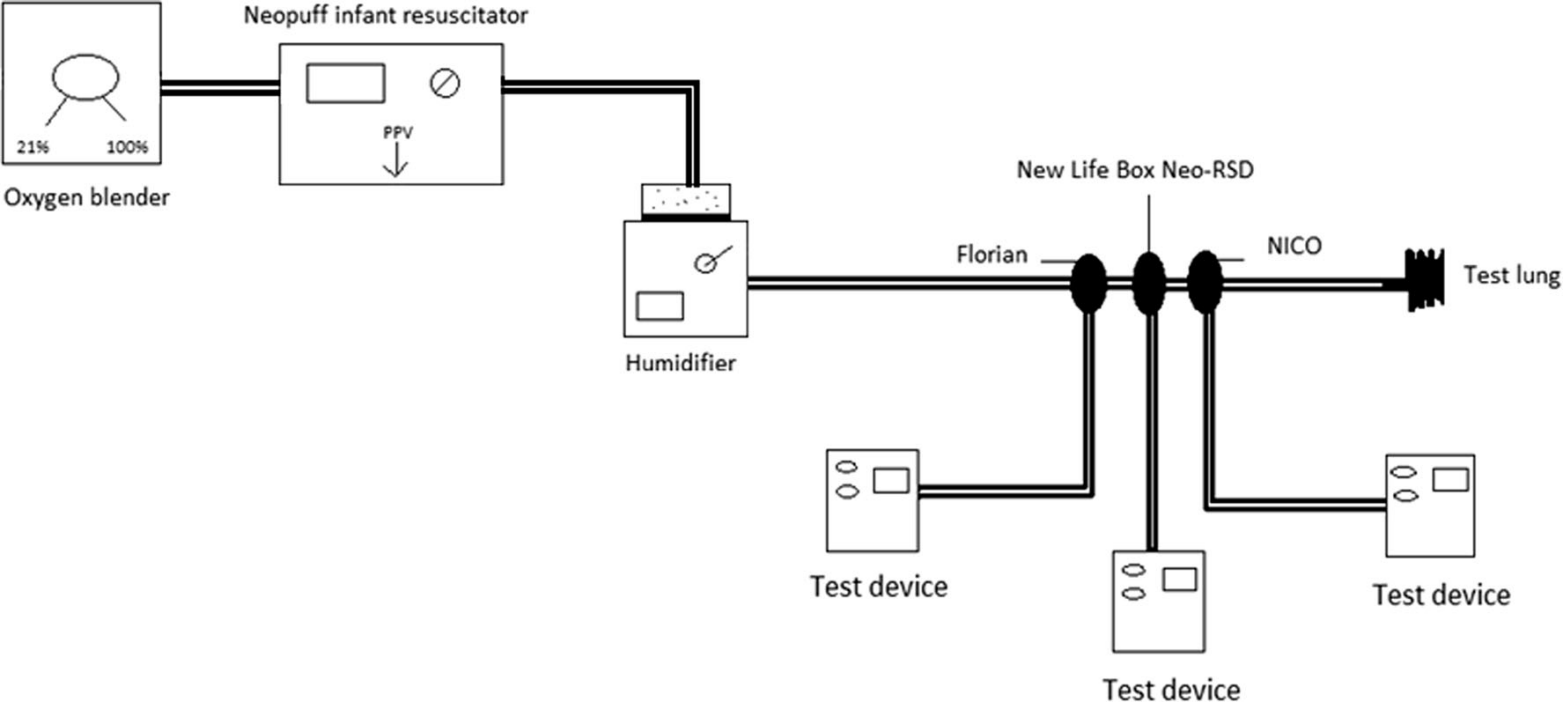
The experimental set-up to test the deviation under different gas conditions

Positive pressure ventilation (50/min) was given using a T-piece ventilator (Neopuff Infant Resuscitator, Fisher & Paykel, Auckland, New Zealand) using a peak inspiratory pressure (PIP) of 25 cm H_2_O and a positive end-expiratory pressure (PEEP) of 5 cm H_2_O [5], and pressures were not changed during the experiment. The T-piece of the ventilator was attached to a 50 mL test lung (Dräger, Lübeck, Germany, compliance of 0.66 mL/mbar) with the three flow probes placed serial between the T-piece and the test lung (Fig. 2). To test the effect of increasing oxygen on the flow measurements, baseline measurements were performed with air and then oxygen was increased to 100 %. To test the effect of changing gas conditions, baseline measurements were performed with cold dry air (24 ± 2 °C) and then humidified heated air (37 °C) was given using a humidifier (MR 850, Fisher & Paykel, Auckland, New Zealand). The system (Fig. 2) was tested to be leak free before the start of each experiment. Four different gas compositions were then tested and compared: 21 % O_2_ dry cold air, 21 % O_2_ humidified heated air, 100 % O_2_ dry cold air, and 100 % O_2_ humidified heated air. The humidifier was placed between the Neopuff and T-piece, and the distance of the tubing to the T-piece was 1.3 m (Fig. 2). During the experiment, the changing in gas condition was repeated 10 times for each flow probe, and two different flow probes were used for each device. Inspired tidal volume was taken as the outcome measurement.

All data were analysed using IBM SPSS Statistics V. 22 (IBM Software, New York, USA, 2012). For the accuracy of the RFMs, we calculated the difference between the known volume given and the volume measured (volume measured–known volume given) and expressed as a percentage of the volume given. The volume differences are presented in mean (SD). As we were only interested in clinically relevant differences, statistical differences are not presented. We considered a volume difference >10 % to be clinically relevant.

## Results

### Accuracy

The average difference from the known applied volume of 10 and 20 mL was smaller using the NLB Neo-RSD compared to both the Florian and the NICO (Table 1). The percentage volume difference remained within the clinically acceptable range of <10 % using all three RFMs except for the NICO when measuring 20 mL (Table 1). While the average volume difference measured with the NLB Neo-RSD and the Florian indicates an overestimation of the tidal volume (Vt), the NICO would underestimate the Vt (Table 1).

**Table 1:** Mean (SD) deviation when measuring 10 and 20 mL in percentages (mean (SD))

RFM	10 mL deviation Vt (%)	20 mL deviation Vt (%)
Florian	8.4 (1.2)	8.4 (0.5)
NLB Neo-RSD	5.8 (1.1)	3.8 (0.8)
NICO	−8.2 (0.9)	−8.7 (0.8)

### Measurements during changing gas conditions

There were small and clinically acceptable changes in the volume differences measured during changes in gas conditions using the NLB Neo-RSD and NICO (Table 2). While tidal volume measurements increased using the NLB Neo-RSD when FiO_2_ and/or air was heated and humidified, there was a decrease using the NICO (Table 2).

**Table 2:** Mean (SD) change in percentage during changing gas conditions

Change	FiO2 0.21–1.0		24 ± 2 °C → 37 °C		FiO2 0.21–1.0 +24 ± 2 °C–37 °C
	Cold dry air	Heated humidified air	FiO2 0.21	FiO2 1.0	
Florian	5.2 (1.2) %	12.2 (0.9) %	12.2 (1.0) %	19.8 (1.1) %	25.7 (1.7) %
NLB	2.0 (1.6) %	3.4 (2.8) %	0.2 (1.9) %	1.1 (2.8) %	3.8 (2.4) %
NICO	−2.3 (0.8) %	0.1 (0.6) %	−5.6 (0.9) %	−3.7 (0.9) %	−5.7 (1.4) %

The largest changes in volume measurements during changes in gas conditions occurred using the Florian. While the increase in volume was acceptable during change in oxygen and cold dry air was used, the increase was above the clinically acceptable range when oxygen was changed when using humidified heated air. When both gas conditions were simultaneously changed, the change in volume measurement increased to 25.7 (1.7)%.

## Discussion

In this bench study, we compared the accuracy of current available RFMs for monitoring respiratory support of infants at birth. We observed that the volume measurements provided by all three RFMs were accurate and within clinically acceptable ranges (<10 %). The smallest volume difference (between known and measured) was observed when using the NLB Neo-RSD. While the Florian and NLB Neo-RSD tended to overestimate the volumes given, the NICO tended to underestimate the tidal volume. Although changing the gas composition influenced the tidal volume measurements in all three devices, only the Florian showed volume differences that we consider are clinically relevant, irrespective of whether the oxygen was increased or temperature and humidification changed. As these changes in gas compositions commonly occur during respiratory support at birth, the caregiver should take this increase into account when the Florian is used for volume measurements.

Using a similar set up, Roske et al. [20] compared RFMs incorporated into mechanical ventilators with the flow meters used in our study. The reported error of the hot wire and variable pressure transducer was comparable to our findings (<10 %), but while we observed an underestimation in the NICO, Roske et al. reported overestimation in the Novametrix, which also uses a fixed orifice along with a differential pressure transducer [20]. The RFMs in the mechanical ventilators were pre-calibrated by the manufacturer, and we speculate that properties, algorithms, data acquisition, and processing have changed the accuracy of the devices in time (16 years). This might explain why the measurements of the Novametrix were different than in the NICO.

The influence of oxygen concentration and gas condition on flow sensor measurements has been described before [3, 22]. Increasing oxygen concentration, humidification, and temperature of the gas increases the gas density, which will influence the resistance at the orifice of the variable and fixed orifice flow meters. The observation that the volume decreased instead of increasing in the NICO most probably indicates that the correction in the algorithm overcompensates for the increase in gas density.

Larger differences in volumes were observed with hot wire anemometer when compared to the differential pressure transducers. As oxygen is a better heat conductor than nitrogen, a higher concentration of oxygen will cause a larger decrease in temperature of the heated wire leading to overestimation of the volume. Although this effect is acceptable when using cold dry air, increasing oxygen concentration led to clinically important differences in the measured volume, particularly when combined with heated humidified air. Similarly, both Roske et al. and Fischer et al. have previously reported that, when using a hot wire flow meter, the error of volume measurement increased (+23.3 (1.7)% and 25 %, respectively) when the oxygen concentration was increased from 21 to 100 % while using heated humidified air [3, 20]. Humidification also causes a larger decrease in temperature of the heated wire leading to a similar effect.

When there is little time to prepare for a preterm delivery, the temperature and humidification of the inspired air may initially be low and may gradually increase following ventilation onset. While the studies reporting volume measurements at birth [14, 15], using a hotwire anemometer, have performed a post analysis correction of the values depending on the gas composition, this is not possible when the volumes are used for direct feedback during resuscitation.

This was an in vitro study where we used a leak free model and ventilated a test lung. For this reason, there was no difference in effect of changing gas condition on Vte and Vti. It is possible that different effects will be observed when measuring tidal volumes during mask ventilation of infants at birth and using a Florian. Most often the expired tidal volume is used for monitoring. When oxygen of the gas is increased, oxygen concentration of the exhaled gas will be lower than the inhaled gas. Nevertheless, the increase in oxygen concentration will be substantially and still have an effect on the expired tidal volumes. In contrast, the effect of heating and humidification could be less on expired tidal volume as exhaled gas is already heated and humidified. However, the larger difference between inspired tidal volume and expired tidal volume would then be misinterpreted as leak.

While testing the accuracy, the volumes were manually given using a glass syringe. Although a timer was used for the respiratory rate, it is possible that flow rates varied, and this likely has influenced the variation in the repeated measurements. For the changing gas condition we used, the Neopuff and the pressures were set only once for the repeated measurements. The measurements of the flow meters were performed in a serial way to ensure that all circumstances, e.g., changes in gas condition, room temperature, and position of the test lung, were identical in every test. Before start of the experiments, we tested the flow probes in different following orders and did not observe any effect of the location of the probes on the measurements. The extra distance of the probe from the humidifier dictated by the following order they were placed at the T-piece would only lead to a very small variation, and the effect on the temperature and humidification would be negligible.

In conclusion, this bench test study has shown that all three RFMs were accurate, and that while differences between the known and measured volumes were detected, these differences were small and clinically acceptable. The NLB Neo-RSD was the most accurate device. Small, but acceptable differences in volumes were measured by the NLB Neo-RSD and the NICO when gas conditions are changed. When gas conditions such as FiO2, humidity, and temperature change during resuscitation, volume measurements of the Florian deviated considerably, especially when heated humidified air is used. These deviations have to be taken into account when interpreting these measurements directly during resuscitation.

## Abbreviations

NLB Neo-RSD: New Life Box Neo-RSD
PEEP: Positive end expiratory pressure
PIP: Peak inspiratory pressure
RFM: Respiratory function monitor
V_t_: Tidal volume
